# PROMOTING HEALTHY AGING THROUGH PRAGMATIC CLINICAL TRIALS

**DOI:** 10.1093/geroni/igz038.919

**Published:** 2019-11-08

**Authors:** Joshua Chodosh, Mary P Cadogan, Jennifer L Martin

**Affiliations:** 1 New York University Langone Health, New York, New York, United States; 2 UCLA School of Nursing, Los Angeles, California, United States; 3 VA Greater Los Angeles Healthcare System, UCLA, North Hills, California, United States

## Abstract

Promoting healthy aging does not end when people enter skilled nursing facilities (SNF) where the demands for clinical and psychosocial care are likely to be greatest. Many chronic conditions present opportunities for better SNF care and thus, healthier aging. Such conditions cannot wait for the often-long path to discovery that is typical of most traditional randomized controlled clinical trials. Conversely, pragmatic clinical trials are real-world investigations that offer the possibility of immediate benefit while answering important research questions. Depression and disrupted sleep are two examples of treatable conditions with opportunities for immediate benefit through pragmatic trials and applied best practices. How best to support best-practice integration has received increasing attention but identifying the most effective strategies continues to evolve. We report two different SNF-mentorship models utilizing Minimum Data Set (MDS) data for depression and environmental (noise-level) data for disrupted sleep, which have supported better SNF practices and presumably, healthier aging.

